# Neuromyelitis Optica Spectrum Disorder: A Rare Case of Isolated Brainstem Syndrome

**DOI:** 10.7759/cureus.5644

**Published:** 2019-09-13

**Authors:** Mai-Lynn Bui, Jordan K Gould, Akshay Mentreddy, Emily Sigsbee, Hector Lalama

**Affiliations:** 1 Neurology, Larkin Community Hospital-South Miami, Miami, USA; 2 Ophthalmology, Larkin Community Hospital-South Miami, Miami, USA; 3 Neurology, Nova Southeast / Larkin Community Hospital-South Miami, Miami, USA

**Keywords:** neuromyelitis optica, neuromyelitis optica spectrum disorders, aquaporin-4 antibodies(nmo antibodies), multiple sclerosis, devic's syndrome

## Abstract

Before 2006, neuromyelitis optica was hallmarked by optic neuritis and transverse myelitis. However, with the discovery of antibodies to water channel aquaporin-4 as a diagnostic criterion, our clinical knowledge of the disease manifested in the creation of new pathologies that fell under the diagnostic umbrella of neuromyelitis optica spectrum disorder (NMOSD). Still, brain involvement of the disease has remained rare, in particular, lesions of the brain stem. Specific to our report is a novel case of NMOSD with intriguing, isolated brainstem findings.

## Introduction

Neuromyelitis optica (NMO), or Devic’s syndrome, is a demyelinating disease hallmarked by two distinct presentations: bilateral optic neuritis and transverse myelitis. While NMO may have a clinical presentation similar to multiple sclerosis (MS), NMO remains an entirely different diagnosis. A review of population-based studies shows a worldwide incidence of about 0.05-0.4 per 100,000 and a prevalence of 0.52-4.4 per 100,000 people. The mean age of onset is reported to be around 32.6-45.7 years of age [1].

In 2004, Lennon et al. documented the discovery of a serum antibody that targets the water channel aquaporin-4. This antibody (AQP4- IgG) proved to be highly specific and instrumental in the diagnosis of NMO [2]. In fact, the discovery of this antibody was key in expanding the diagnostic criteria for NMO in 2006 [3]. Eventually, the term neuromyelitis optica spectrum disorder (NMOSD) was created in 2007 to encompass patients with positive serology to AQP4-IgG [4].

Historically, one of the clinical criteria for the diagnosis of NMO was a lack of brain involvement on magnetic resonance imaging (MRI) [5]. However, more and more studies have cited the involvement of the brain. While brain involvement may seem common, brainstem involvement remains relatively quite rare. In fact, only about one-third of NMOSD presents with brainstem involvement [6]. The presentation of brainstem symptoms due to the involvement of the area postrema of the medulla can lead to the initial presentation of sometimes intractable nausea and vomiting with associated medullary lesions on MRI in 16% to 43% of patients with NMOSD [7]. Specific to our case is a rare presentation of NMOSD isolated to the brain stem.

## Case presentation

Our patient was a 32-year-old female, with no known significant past medical history, who presented to the hospital for a total of two different admissions. On the first admission, our patient presented with initial complaints of intractable nausea and vomiting, odynophagia with hypersalivation, generalized right-sided weakness, left-sided numbness/tingling, sensory disturbance to the face and tongue, and dysfunction leading to dysphagia and difficulties with speech. Symptoms had begun within the past month and had never occurred prior. The patient was originally healthy overall, and reported no known significant or contributory family history, endorsed no past surgical history, had no known allergies, and did not take any home medications. There was no history of alcohol or substance abuse.

Eventually, neurology was consulted and evaluated the patient on admission for generalized weakness. On examination, the patient was found to be alert and oriented to person, place, and time with no noted global or expressive aphasia, no dysarthria appreciated, with the ability to answer simple questions appropriately and follow simple commands. Her cranial nerves II-XII were grossly intact. Motor function, mass, and bulk were age-appropriate, without atrophy. Tone was appropriate, with no spasticity, no rigidity, no flaccidity, no ankle clonus, no fasciculations, no tremor. Findings were: deep tendon reflexes (DTRS): 3+ globally in bilateral biceps, triceps, brachioradialis, patellar, Achilles; Babinski’s were downgoing bilaterally while Hoffman's were negative bilaterally. Strength showed no Hoover sign, left elbow flexors/extensors (5/5), left shoulder (5/5), left finger flexor/extensors (5/5), right elbow flexors/extensors (4/5), right shoulder abduction/flexion (4/5), right finger flexors/extensors (4/5), left lower extremity (LE) hip flexion/extension (5/5), left knee flexion/extension (5/5), left ankle flexion/extension/eversion/inversion (5/5), right LE hip flexion/extension (4/5), right knee flexion/extension (4/5), and ankle flexion/extension/eversion/inversion (5/5). Coordination was intact, as the patient was able to perform bilateral finger to nose without dysmetria and performed bilateral heel to shin adequately; rapid alternating movements were intact and symmetric, no dysdiadochokinesia was present. Sensory function was intact, as temperature/pain/proprioception/vibration and light touch were grossly normal/intact in the bilateral upper and lower extremities, face, and torso. Rhomberg was negative. The patient was able to display adequate casual gait with stride length and arm swing, adequate tandem gait, heel walk, and toe walk.

Further workup was performed to investigate possible autoimmune causes of weakness/paresthesias. CT brain without contrast was performed, which showed unremarkable results. Labs reviewed at that time revealed erythrocyte sedimentation rate (ESR) 28, C-reactive protein (CRP) within normal limits (WNL), B12 223 low normal, folate 20.6 WNL, thyroid-stimulating hormone (TSH) .63 WNL, electrolytes unremarkable, calcium 9.1 WNL, rapid plasma reagin (RPR) nonreactive, magnesium 2.78, phosphate 4.1, creatinine kinase (CK) WNL, iron panel with total iron-binding capacity (TIBC) mildly low but otherwise ferritin and iron WNL. Results also showed antinuclear antibody (ANA) positive, anti-dsDNA negative, anti-histone negative, anti-JO1, anticentromere negative, and AchR negative. An electromyogram (EMG)/nerve conduction velocity (NCV) study was performed of the bilateral lower extremities and upper extremities, tongue, sternocleidomastoid, and trapezius for the indication of right-sided extremity weakness/tremor and tongue weakness/tremor. The study showed no evidence of neuropathy or myopathy. The patient was discharged by the primary team following an improvement in her symptoms and an unremarkable workup.

In about three weeks time, the patient was readmitted to the hospital for similar complaints as before. Of note, on the second admission, the patient presented with new-onset postural instability, which hindered her ability to ambulate. The patient also complained of double vision, difficulty swallowing, and generalized weakness. On examination, the patient remained alert and oriented to person, place, and time, with no global or expressive aphasia, and no dysarthria was appreciated. The patient answered simple questions appropriately and followed simple commands. Cranial nerves II-XII were grossly intact, however, pronounced lateral nystagmus was appreciated in both eyes to the left and right. Motor function and tone were within normal limits. Strength in all four extremities was preserved. The patient was with noted hyperreflexia in the bilateral lower extremities. Coordination and sensation were intact. Gait was poor with short stride length, in addition to truncal dysmetria and postural imbalance.

**Figure 1:**
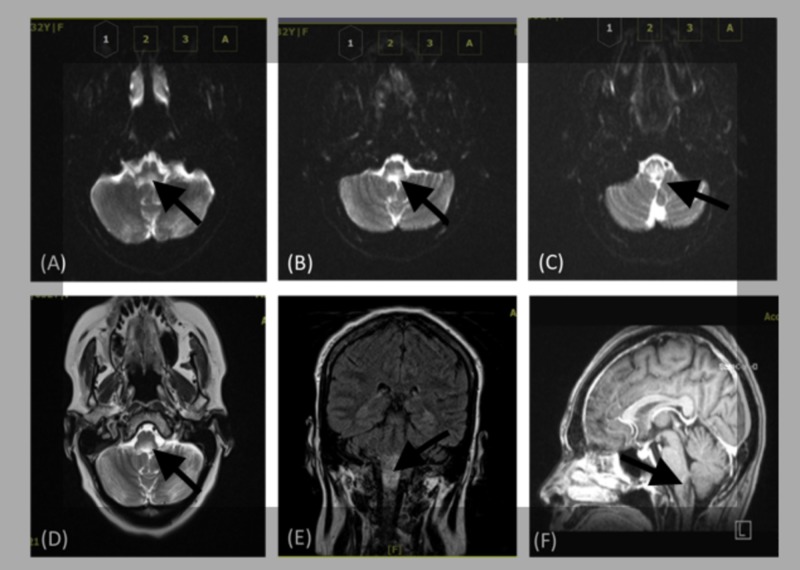
MRI T2 brain axial view with visualization of (see arrows corresponding to respective areas of interest) (A) lower medulla at the level of the cerebellum. Note a hyper-attenuated lesion situated at the dorsal medulla; (B) medulla and cerebellum at the lower maxillary level. Note the continuation of the hyper-attenuated lesion situated in accordance with the previous lesion, localized to the dorsal medulla; (C) medulla and cerebellum at the upper maxillary level. Note also the continuation of the hyper-attenuated lesion situated in accordance with the previous lesion, localized to the dorsal medulla; (D) MRI T2 FLAIR brain axial view with visualization of the lower medulla at the level of the cerebellum. Note a hyper-attenuated lesion situated at the dorsal medulla with increased T2 signal and mild diffuse and heterogeneous enhancement; (E) MRI T1 brain coronal view depicting a 1 x 2.5-centimeter hyper-attenuated, isolated lesion at the dorsal medulla. (F) MRI T1 brain sagittal view depicting a 1 x 2.5-centimeter isolated lesion at the dorsal medulla. There was no definite enlargement of the bones or the upper cervical cord. There were no definite areas of abnormal enhancement. There were no areas of restricted diffusion. MRI: magnetic resonance imaging; FLAIR: fluid-attenuated inversion recovery

An MRI brain w/wo contrast was ordered with a specific focus on the visualization of the brainstem (Figure 1). Results yielded a hyperintense lesion identified at the lower medulla, which was suspicious for a malignant process vs a rapidly demyelinating lesion.

Labs reviewed on most recent admission revealed vitamin B12, folate, TSH, magnesium, and phosphate within normal limits. Prior labs for an autoimmune workup from the previous visit were reviewed as well. RPR from the previous visit was non-reactive. A paraneoplastic panel (Anti-Hu, Anti-Yo, Anti-Ri) was unremarkable. A lumbar puncture was performed, and the cytology of CSF sent for glucose, protein, venereal disease research laboratory (VDRL), gram stain, oligoclonal bands, myelin basic protein, immunoglobulin G (IgG) synthesis rate, cell count with differential, cytology to check for cancer cells, fungal stain, cryptococcal antigen/antibody, India ink, and NMO antibody. Results from LP are shown in Table 1. With NMO IgG antibody increased at 4.1 and all other tests unremarkable, the patient was diagnosed with NMOSD. The patient was started on intravenous (IV) Solu-Medrol 500 mg q12h seven days. The patient was also scheduled for three days of plasmapheresis in addition to the high-dose steroid therapy. On outpatient follow-up appointment, the patient stated significant improvement to her previous symptoms upon last hospital admission. Eventually, the patient was discharged and continued on immunosuppressant therapy of Imuran 50 mg daily and prednisone 10 mg daily. The patient was instructed to follow up with neurology in an outpatient setting.

**Table 1:** CSF Results * denotes an elevated value. Our patient had elevated WBC and NMO IgG antibody levels consistent with a diagnosis of NMOSD. The patient also had an elevated CMV IgG but negative CMV IgM, indicating a history of previous CMV infection. CSF: cerebrospinal fluid; CMV: cytomegalovirus; WBC: white blood cell; RBC: red blood cell; IgG: immunoglobulin G; IgM: immunoglobulin M; NMOSD: neuromyelitis optica spectrum disorder

CSF Protein	28 mg/dL
CSF Glucose	93 mg/dL
CSF WBC	16 cells/microL
CSF Cell Count with Differential.	
Color	Colorless
RBC	2 cells/microL
WBC	16* cells/microL
Lymphocytes	97 cells/microL
Monocytes	3 cells/microL
Appearance	Clear
Protein	28 mg/dL
Glucose	93 mg/dL
Cryptococcal Antigen	Negative
Gram Stain	No organisms grown or WBCs within 24 hours.
NMO IgG Antibody	4.1* U/mL
CSF Cytology for Malignant Cells	Negative
West Nile IgM	Negative
Quant Gold Antibody	Negative
CMV IgM	< 30 U/mL
CMV IgG	>10* U/mL

## Discussion

At initial presentation, our patient exhibited intractable nausea and vomiting, odynophagia with hypersalivation, generalized right-sided weakness, left-sided numbness and tingling, sensory disturbance of the face, and tongue dysfunction, leading to dysphagia and speech difficulties. On the second admission, there was a recurrence of these symptoms. In addition, our patient exhibited poor gait with truncal dysmetria and postural imbalance, diplopia, lateral nystagmus, and generalized weakness. These findings, indicative of acute brainstem syndrome, in addition to MRI brain demonstrating a hyperintense lesion at the lower medulla and CSF demonstrating elevated APQ4-IgG met the diagnostic criteria for NMOSD [6].

Presentation of brainstem symptoms due to the involvement of the area postrema of the medulla can lead to an initial presentation of sometimes intractable nausea and vomiting with associated medullary lesions on MRI in 16% to 43% of patients with NMOSD7. This case report highlights the importance of recognizing intractable nausea and vomiting as a possible initial presentation of NMOSD. This case was unique in that our patient exhibited strictly brainstem-related findings.

In a study conducted by Apiwattanakul et al., it was demonstrated that in 12% of AQP4-IgG positive patients from the Mayo Clinic in 2005, the initial presenting symptom of NMOSD was intractable vomiting [7]. In a summary of the present knowledge of typical and atypical manifestations of NMOSD by Jinming et al., it was emphasized that the traditional manifestations of NMOSD are acute myelitis and optic neuritis, with or without AQP4-IgG [8], not typically an isolated acute brainstem syndrome marked by intractable nausea and vomiting. It is thus necessary to continue to examine the wide range of typical and atypical presentations of NMOSD in order to better and more quickly identify and treat the disease.

## Conclusions

This case featured a 32-year-old female who demonstrated a rare, isolated acute brainstem syndrome of NMOSD with the less commonly reported symptoms of facial palsy, vestibular ataxia, and other cranial nerve abnormalities. Her workup over the course of her care, which included neuroimaging of MRI brain w/wo contrast yielded a hyperintense lesion identified at the lower medulla, in addition to a lumbar puncture with cytology of CSF consistent with NMO IgG antibody increased at 4.1, confirming a diagnosis of NMOSD. To our understanding, this is a novel presentation of a rare case of NMOSD isolated to the brain stem within an already diverse spectrum of pathologies of this disease.
